# Stacks of Azobenzene Stars: Self-Assembly Scenario and Stabilising Forces Quantified in Computer Modelling

**DOI:** 10.3390/molecules24234387

**Published:** 2019-11-30

**Authors:** Vladyslav Savchenko, Markus Koch, Aleksander S. Pavlov, Marina Saphiannikova, Olga Guskova

**Affiliations:** 1 Dresden Center for Computational Materials Science (DCMS), Technische Universität Dresden, 01062 Dresden, Germany; vladyslav.a.savchenko@googlemail.com (V.S.); grenzer@ipfdd.de (M.S.); 2 Institute Theory of Polymers, Leibniz Institute of Polymer Research Dresden, Hohe Str. 6, 01069 Dresden, Germany; koch-markus@ipfdd.de; 3 Department of Physical Chemistry, Faculty of Chemistry and Technology, Tver State University, Sadovyj per. 35, Tver 170002, Russia; as_pavlov@list.ru

**Keywords:** azobenzenes, self-assembly, cooperativity, hydrogen bonding, computer simulations

## Abstract

In this paper, the columnar supramolecular aggregates of photosensitive star-shaped azobenzenes with benzene-1,3,5-tricarboxamide core and azobenzene arms are analyzed theoretically by applying a combination of computer simulation techniques. Without a light stimulus, the azobenzene arms adopt the trans-state and build one-dimensional columns of stacked molecules during the first stage of the noncovalent association. These columnar aggregates represent the structural elements of more complex experimentally observed morphologies—fibers, spheres, gels, and others. Here, we determine the most favorable mutual orientations of the trans-stars in the stack in terms of (i) the π–π distance between the cores lengthwise the aggregate, (ii) the lateral displacements due to slippage and (iii) the rotation promoting the helical twist and chirality of the aggregate. To this end, we calculate the binding energy diagrams using density functional theory. The model predictions are further compared with available experimental data. The intermolecular forces responsible for the stability of the stacks in crystals are quantified using Hirshfeld surface analysis. Finally, to characterize the self-assembly mechanism of the stars in solution, we calculate the hydrogen bond lengths, the normalized dipole moments and the binding energies as functions of the columnar length. For this, molecular dynamics trajectories are analyzed. Finally, we conclude about the cooperative nature of the self-assembly of star-shaped azobenzenes with benzene-1,3,5-tricarboxamide core in aqueous solution.

## 1. Introduction

In recent years, azobenzene-containing molecules (azos), which are reversibly responding to the light stimulus, have been widely applied in science and engineering to construct materials with various photosensitive characteristics [1,2,3,4,5]. Among others, star-shaped azos, i.e., multi-photochromic compounds with azobenzene moieties attached to the central unit as arms, have received considerable attention in the research of the past decade. On one hand, such molecules combine mesomorphism and photoswitching [6,7,8], they can more effectively reply to the light stimulus (for example, during inscribing a surface relief grating) as compared to monomeric azos. On the other hand, they can be readily synthesized and purified, be either evaporated or cast from solution, build stable amorphous phases and exhibit more uniform physical properties. There is no dilution of the chromophores or any effects coming from entanglements unlike azo–photochromic polymers and composite polymer systems [9,10]. Star-shaped azos are also different from linear or cyclic azo *n*-mers with the same number of azo-fragments (trimers, tetramers, etc.). First of all, this is manifested in different supramolecular structures the stars and the linear or cyclic [11] *n*-mers can build. Moreover, the branching results in lower melting temperatures, and lower viscosity [6,12,13], meaning that these properties are topology-dependent.

There are several classifications of star-shaped azos: they may differ in (i) the number of arms (three [9,10,14,15,16,17,18,19,20], four [7,21,22], six [23,24,25], nine [26]), (ii) in their centres (nitrogen [9,19], phosphorus [27], silicon [28] or carbon atoms [21,22], benzene [14,16,20,29,30,31,32], polycyclic aromatic hydrocarbons [25,33], heterocyclic hydrocarbons [34,35,36,37], some chiral groups [6] or bio-active residues [38]), (iii) in the way azobenzenes are attached to the centre (covalently or noncovalently [39]), (iv) in their conformational rigidity (flexible or rigid), and (v) in their overall geometry (quasi-planar or 3D). Both the conformational rigidity and the shape of the star are closely related to the chemical nature of the star core. For instance, the planar conjugated fragment in the absence of any fatty linkers between the centre and the azobenzene arms supports the planarity of the star and its rigidity, and vice versa, long hydrocarbon linkers provoke its conformational flexibility [8,30,31].

**Figure 1:**
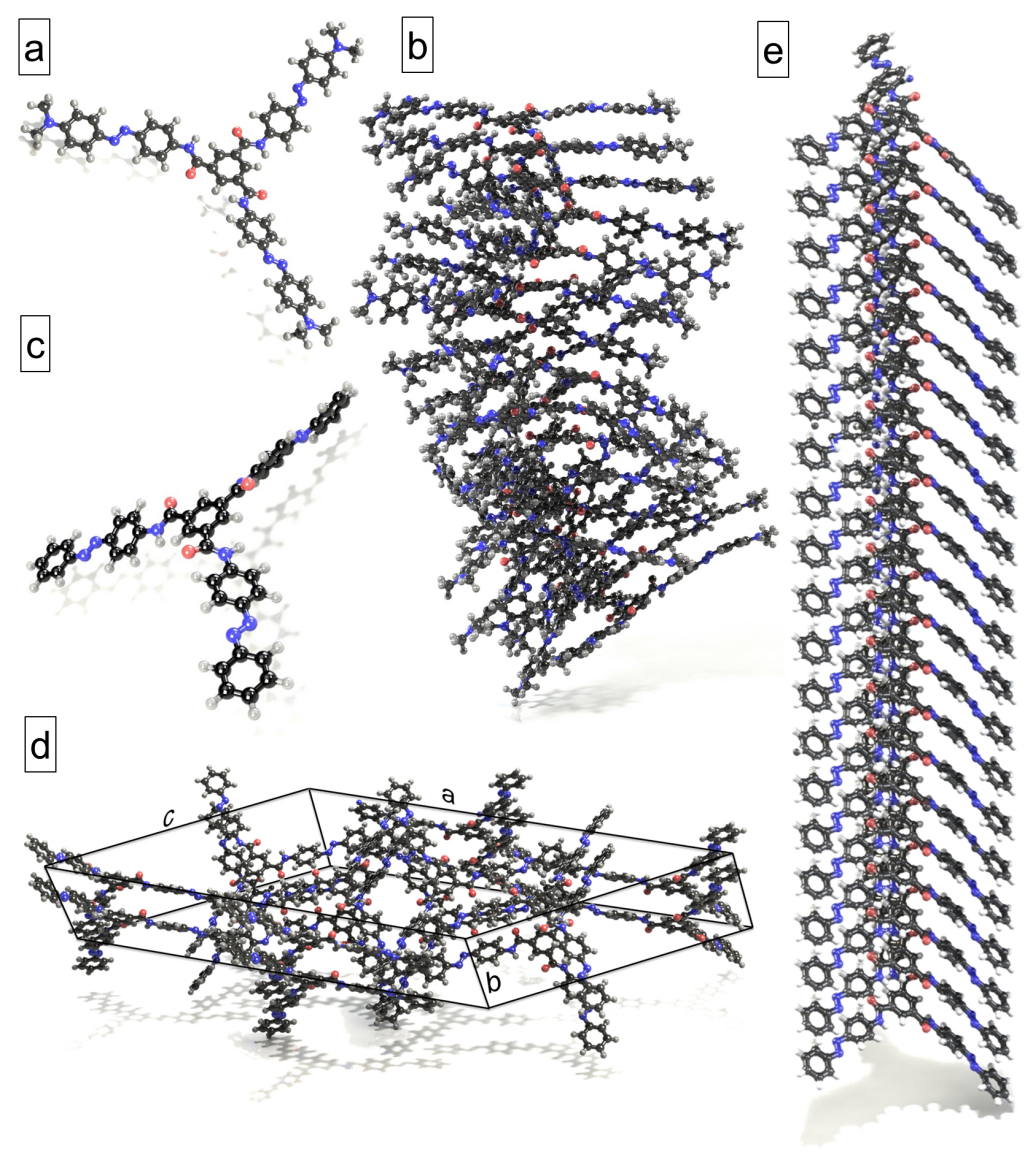
The object of the study: (**a**) the azo–benzene-1,3,5-tricarboxamide (BTA) star with benzene-1,3,5-tricarboxamide core and three azobenzene arms. Each arm carries the *N*, *N*-dimethylamino group. Here, the optimized geometry is shown [14]. A snapshot of the columnar aggregate with *N* = 20 from MD trajectory [29] is illustrated in panel (**b**). The azobenzene star studied experimentally by Lee et al. [17] is depicted in panel (**c**). The conformation of this star is the same as in the crystalline structure, shown in panel (**d**). Here, *a* = 59.37 $\overset{˚}{A}$, *b* = 9.42 $\overset{˚}{A}$ and *c* = 39.95 $\overset{˚}{A}$; α = β = 90° and γ = 103.16°. The original unit cell [17] is multiplied twice along the *b*-direction to illustrate the stacking of the stars. The panel (**e**) shows the stack made of 20 such molecules. The carbon, hydrogen, oxygen and nitrogen atoms are colored dark grey, light grey, red and blue spheres, respectively.

In this work, we investigate aqueous solutions of azobenzene star molecules with a planar benzene-1,3,5-tricarboxamide (BTA) core and three azo arms (azo–BTA star, see Figure 1a). This star is rigid and quasi planar in its all-trans state, as recently confirmed by us [14,29]. Its BTA core is a well-known supramolecular entity in colloid science, used for the fabrication of dynamic and responsive materials [40,41,42,43,44,45,46]. The interactions between BTA molecules are inherently dynamic and involve hydrogen bonding between three amide groups, π–π stacking interactions between benzene cores, and hydrophobic forces originating from the arms attached to the centre. Tuning these interactions and applying a light stimulus, one can control the colloidal assembly of such molecules in solution.

As literature survey shows, azo–BTA stars that differ from the one shown in Figure 1a only in the *para*-substituents at the star periphery, demonstrate light-controlled self-assembly and actuation [17,26,47]. For example, Choi et al. observed the construction of nanocolumns and further lateral self-assembly into the low-ordered hexagonal columnar liquid crystal phase or lamellar-columnar crystals [26]. Not only the shape of supramolecular assemblies but also their color are shown to be remote-controlled by light [26]. Lee et al. have studied the solvent dependent generation of microfibers, gels, and hollow spheres in aqueous organic solvent systems and their photoinduced and reversible phase transitions upon UV-Vis light illumination [17]. One new study demonstrated the perpetual columnar self-assembly of azo–BTA discotic mesogenic blocks at room temperature which hold together despite light stimulation [48]. Another interesting example of self-assembly of nonchiral azo–BTA stars into mesophases with photo-switchable chirality was demonstrated at a recent time by Choi et al. [49].

Light-sensitivity of azo–BTA stars has been used for the fabrication of lyotropic liquid crystalline alignment media [50], for the light-controlled synthesis of gold nanoparticles [51], for the inscription of surface relief [10,52,53] and holographic [32] gratings, as well as for patterning and molecular alignment [30,31,54]. In a recently published review [55], it has been highlighted that crystalline phases consisting of azo–BTA stars show remarkable promise to be applied as photomechanical crystalline nanowire actuator utilized for the gripper of a micropipette [18]. Moreover, azo–BTA stars, owing to their chemical structure and depending on the experimental conditions, show the ability to form homogeneous molecular glasses [10,32,52,53] (an amorphous phase), to build a liquid-crystalline phase [26,30,31,54], and to self-assemble into ordered crystalline arrays [17,18].

As pointed out in numerous publications devoted to the self-assembly of azo–BTA stars, the hydrogen bonding and π–π stacking between the neighboring star centers along the columns play an essential role in this system. These two interactions are responsible for assembling a single structure from very first aggregates, that appear at the initial stage of the ordering process. The question of quantifying these stabilizing forces is open and is, therefore, one of the goals of this study.

Our attention will also be given to the self-assembly mechanism of azo–BTA stars. According to the latest scientific ideas in this field, three scenarios of the supramolecular assembly are distinguished: cooperative, isodesmic and ring-chain mechanisms [56]. Two of them, cooperative [43,44] and isodesmic [40,41,42], were identified for BTA-centered molecules. In a recent review, the “structure–mechanism” correlation has been established and the ordering behaviors of BTA-containing derivatives were placed on the so-called cooperativity scale [45]. Thus, the analysis of the signatures of self-assembly is the second aim of this study.

The remainder of this article is divided into three sections. The next chapter contains the discussion of the results and consists of three sub-chapters: The binding energy diagrams calculated for the azo–BTA dimers are discussed first, followed by the Hirshfeld surface analysis of the crystalline azo–BTA star phase found in the literature [17], and then concluded by the quantification of the signatures of cooperative growth of the star-shape azobenzenes stacks. This section is followed by conclusions and how the results can be interpreted in the perspective of previous studies and of the working hypotheses. Lastly, we give an overview of the models and computer simulation techniques applied in the study.

## 2. Results and Discussion

### 2.1. Binding Energy Diagrams

We begin the description of the results with a discussion of the dimers made of azo–BTA stars (Figure 1a). The orientation of the molecules in dimers is parallel, and hence their structure can be quantified by their segregation meaning that they are placed one above another. The binding energy (*E*_B_ in kJ mol^−1^) for the molecular dimers and the assemblies of larger *N* (see below) is estimated as

(1) $$E_{B}=\frac{E_{N}-N\;E_{1}}{N-1},$$

where *N* is the assembly size, *E*_N_ is the energy of the column of size *N*, and *E*_1_ is the energy of the monomer, i.e., the azo–BTA star. More details can be found in Section 4.

**Figure 2:**
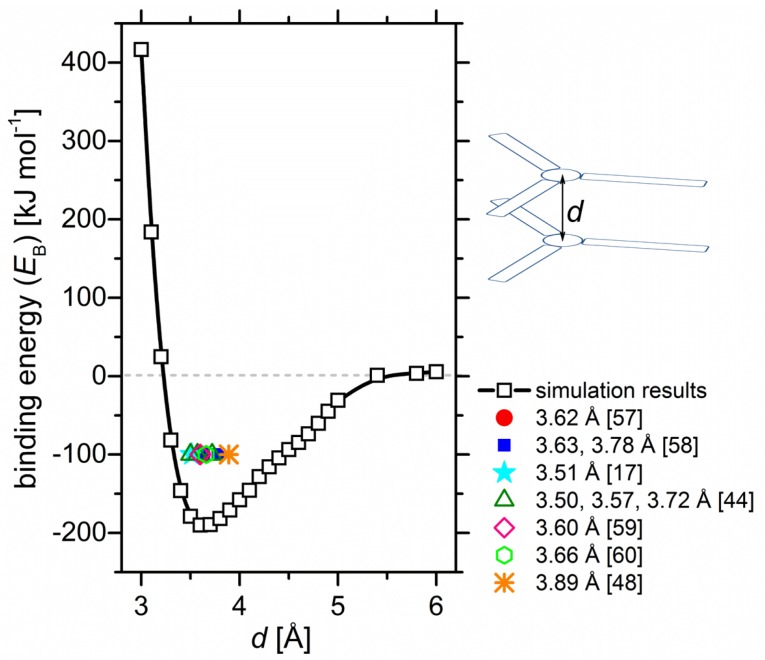
Binding energy *E*_B_ depending on the π–π distance *d* between the star cores in the dimer. The literature values [17,44,48,57,58,59,60] of the π–π distances of BTA-cored stars (in some of these publications called dendrimers) with various arms are shown as symbols with ordinate *E*_B_ = −100 kJ mol^−1^ for convenience. In case of the slipped stacks from the experimental data [17], the stars were shifted or rotated so that they segregated in order to evaluate a true π–π distance between their centres. The definition of *d* is given by the sketch.

The varied parameter for calculation of the binding strength is the distance *d* between the centers of the BTA cores, as schematically shown in Figure 2. The selected *d* values are ranging from 3 to 6 $\overset{˚}{A}$ with a step of 0.1 $\overset{˚}{A}$ which is near experimentally observed π–π distances. The task here is to determine the most energetically favorable distance between the star cores in the column. This task sounds rather trivial, but on one side, the knowledge of this distance is necessary for subsequent simulations, and on another side, the experimental benchmarking using data from the literature allows us to learn about the accuracy of the computer-based research methods.

The analysis of the experimental data [17,48,57,58,59,60] on the ordered phases of various BTA–cored three-armed stars reveals that the intermolecular separations in the stacks range from 3.51 to 3.89 $\overset{˚}{A}$ for the molecules with different periphery around the star center. Apparently, there is no clear relationship between the distance *d* and the bulkiness of the star arms. For example, for almost the same azobenzene [17] and alkoxy azobenzene [48] BTA–stars, the values differ by 0.47 $\overset{˚}{A}$, whereas the π–π stacking separations are nearly equal to 3.6 $\overset{˚}{A}$ for shorter (2-methoxyethyl [57] and carboxymethyl [60]) and longer/bulkier arms (linear and branched C_8_–C_11_ alkyls [58], Glu-based oligopeptides [59]). The reason may lie in the many possibilities the molecules can be assembled in the ordered phases: The stars can build segregated columns, be rotated around the core in the star plane or shifted laterally along the columns.

We would like to stress here that the values found in experimental investigations of similar compounds belong to the range of negative binding strengths. The strongest coupling between the azo–BTA stars in computer simulations corresponds to *d* = 3.6 $\overset{˚}{A}$, which is in the range of the experimental values discussed above [17,48]. The energetics of the binding is ca. −200 kJ mol^−1^. For example, the binding of methyl-substituted BTA proceeds with the energy of ca. −150 kJ mol^−1^, as evaluated in DFT calculations with counterpoise correction by Filot et al. [44]. Taking into account additional dispersion interactions coming from azobenzene arms, the estimated energy for the azo–BTA star seems reasonable. Increasing *d* up to 3.9 $\overset{˚}{A}$ has only little effect on the binding of the stars in the column, indicating that the separations 3.5 ≤ *d* ≤ 3.9 $\overset{˚}{A}$ could be realized without significant energy losses (the variations in *E*_B_ are 1–20 kJ mol^−1^).

**Figure 3:**
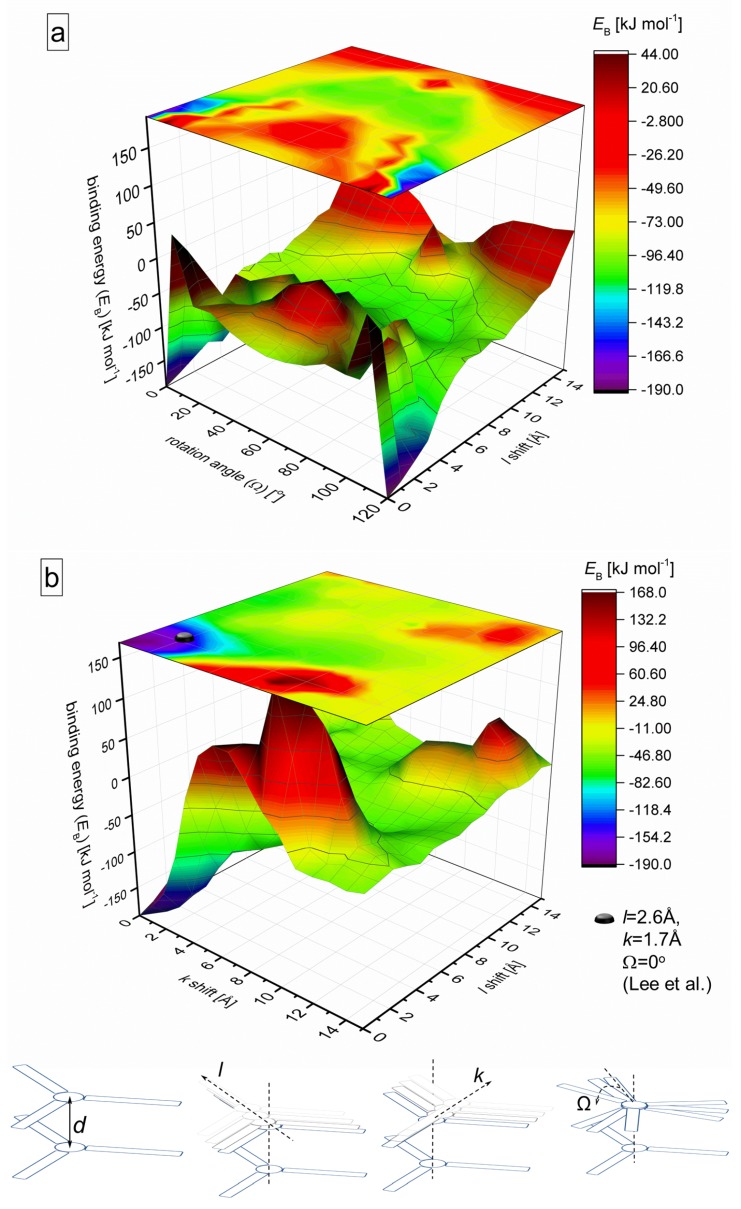
Binding energy *E*_B_ depending on the *l* shift and the rotation angle Ω in star dimer (*d* = 3.6 $\overset{˚}{A}$, *k* = 0 $\overset{˚}{A}$) (**a**) and as a function of *l*, *k* shifts (*d* = 3.6 $\overset{˚}{A}$, Ω = 0°) (**b**). The experimental coordinates [17] are shown as a black bullet on the top projection (**b**). The definitions of *d*, *k*, *l* and Ω are given on schemes at the bottom.

As mentioned above, not only the segregated stacks were found in experiments or predicted theoretically, but also molecules rotated around the star center [59,60] leading to helically twisted arrangements [42,44,49,58,61] in the columns or shifted laterally along the stack [17]. Therefore, as the next step of the study, diagrams are constructed showing the binding energy *E*_B_ depending on the lateral shifts *l*, *k* and the twist angle Ω in star dimer at *d* = 3.6 $\overset{˚}{A}$ (Figure 3). The *l*-shift assumes the displacement of one star in dimer along one of the arms, whereas the *k*-shift occurs in between two arms perpendicular to the *l*-shift. The angle Ω, defined as the rotation angle of the upper star, is shown schematically in Figure 3. Such a systematic rotation of the consecutive stars along the column leads to the formation of the helical structures found in the literature for BTA-centered molecules [44,58,62,63,64]. It was discussed that this twisting allows the binding of the stars through hydrogen bonding, in which all three amide groups of the BTA are involved. Moreover, it reduces possible steric clashes of the arms.

The diagrams provide a graphical visualization of the effects of the variables *l*, *k* and Ω on the intermolecular coupling, i.e. the binding strength. Both panels of Figure 3 illustrate that the most stable stacking is achieved if the stars are perfectly segregated, or slightly shifted/rotated. Concerning the rotation of the stars in the stack (Figure 3a), which is favorable for the triple helical hydrogen bonding, we can conclude that rotation is strongly suppressed in the perfectly stacked columns at the Ω values of 5–10°, where the binding proceeds with positive energy. The coupling becomes more developed for the stacks with shifted molecules along one of the arms and for rotation angles Ω > 10°, meaning that the energy is always negative. For example, for small *l*-shifts and Ω = 0° there is a relatively large area with energies ranging from −50 to −30 kJ mol^−1^ (marked with red color), and another big area for angles 10 < Ω≤ 60° with energies from −120 to −70 kJ mol^−1^ for *l*-shifts from four to eight $\overset{˚}{A}$ (marked with green color).

Let us now compare the literature data with our predictions. The most common rotational angle between consecutive unmoved (*l* = *k* = 0) BTA-centred monomers is 60–65° [44,58,62,63,64]. The calculations show that the binding energy corresponding to these coordinates is about −73 kJ mol^−1^ for the azo–BTA star (marked with yellow color). Interestingly, around Ω = 60° a local minimum on this symmetrical diagram is observed. Smaller rotational angles are also found in literature, for instance, 13–16° for the consecutive stars [40].

The second panel (Figure 3b) shows a wide range of binding energies as well. A combination of small *k* and *l* up to 3–4 $\overset{˚}{A}$ is characterized by the strongest binding. The experimentally found coordinates [17] belong to this global minimum, which coincides with our predictions. The most disadvantageous mutual orientation of stars in the stack is at *k* = 8–10 $\overset{˚}{A}$ and *l* = 0–2 $\overset{˚}{A}$, which is caused by the steric repulsion of the molecular centre and dimethylamino groups at the arm ends. In both panels, the dramatic shifts weaken the coupling considerably.

Note here, that the binding diagrams are calculated with the assumption that the molecular conformation (in our case, almost planar geometry of the star after its optimization [14]) does not alter upon dimerization, translation or rotation. On the contrary, the formation of hydrogen bonds requires not only the rotation of the stars but also the alteration of the planarity of the BTA cores. More realistic binding models will be considered further when we will discuss the mechanisms of self-assembly of azo–BTA stars in aqueous solution.

### 2.2. Analysis of the Trans-Azo–BTA Star Crystal

In this section, we move from the simplified dimer representation to a more complex organization of the azo–stars, as experimentally investigated by Lee et al. [17]. The crystalline phase found in literature [17] is shown in Figure 1d.

**Figure 4:**
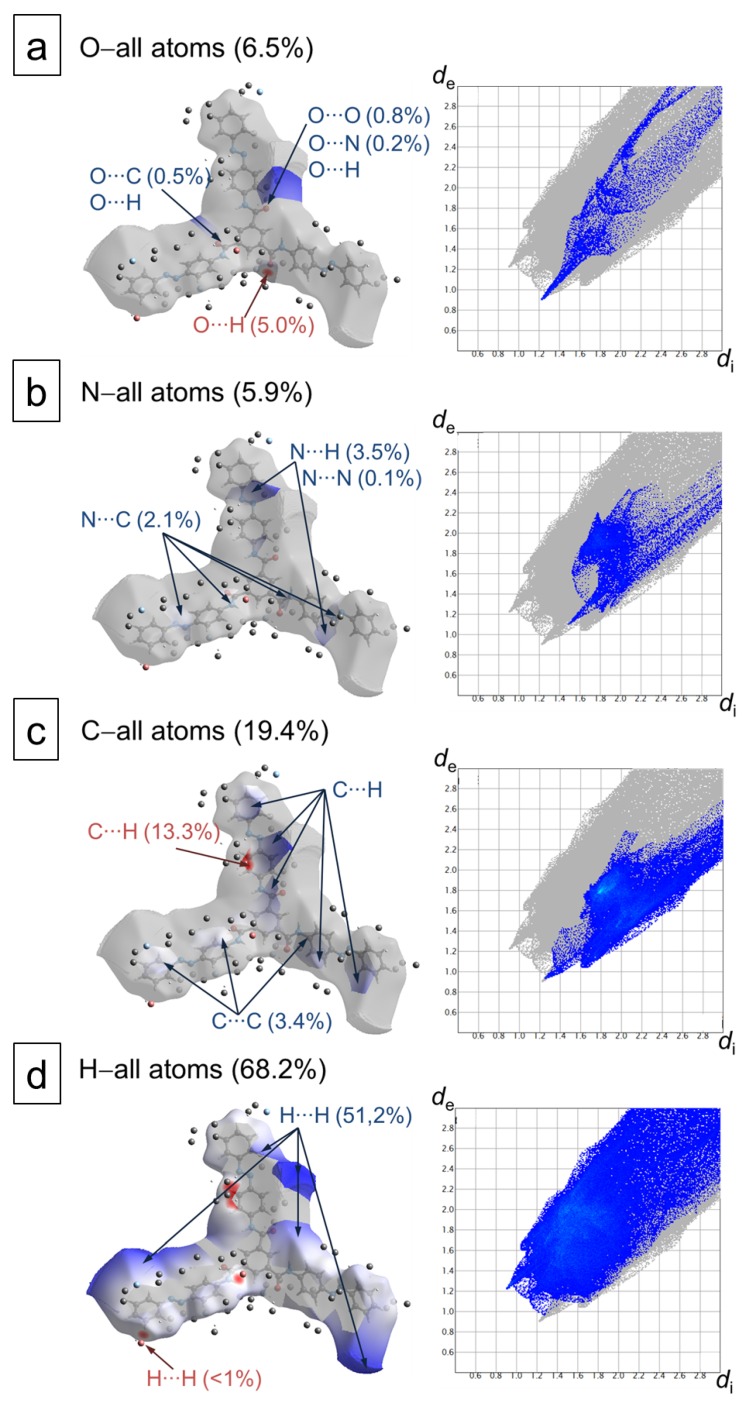
Hirshfeld surface analysis [65] of the azo–BTA star crystal studied by Lee et al. [17] and the fingerprint plots [66] (*d*_e_-*d*_i_ diagrams) of intermolecular interactions between oxygen (**a**), nitrogen (**b**), carbon (**c**), hydrogen (**d**) and other atoms in the molecular crystal. The percentage of the corresponding interaction is given in parentheses. The Hirshfeld surface is shown as a semitransparent surface with a reference molecule inside. The contacting atoms from the neighboring stars in the crystal are shown as beads. For the color scheme see Figure 1.

We are most interested in studying the intermolecular contacts, holding together or destabilizing the stacks of stars in the crystal. To probe them, we use the Hirshfeld surface analysis [65] (Figure 4, left column) and plot the *d*_e_-*d*_i_ diagrams (Figure 4, right column). The color scheme of the surface codes the sign of the normalized contact distance *d*_norm_ defined in terms of *d*_e_, *d*_i_ and the van der Waals (vdW) radii of the atoms (see Section 4 for more detail).

Here, $d_{e}$ and $d_{i}$ are the distances from the Hirshfeld surface to the nearest atom outside (external) and inside (internal) the surface, correspondingly. The color of the Hirshfeld surface changes from red (showing the distances shorter than the sum of vdW radii) through white (distances are equal to the sum of vdW radii) to blue (distances are longer than the sum of vdW radii). The fraction of the corresponding atomic contacts is also given in percent.

For example, the contacts between oxygen and hydrogen atoms (Figure 4a) are shorter than the sum of vdW radii, indicating the hydrogen-bonded arms along the molecular stack. The distance of the separation between two participating atoms is 2.24 $\overset{˚}{A}$. Two sharp lateral spikes with a minimum *d*_e_ + *d*_i_ ≈ 2.24 $\overset{˚}{A}$ correspond to the O⋯H contacts (Figure 4a). The O⋯H contacts comprise 5.0% of the surface.

The Hirshfeld surface provides evidence for C–H⋯π interactions (between hydrogen atoms of the phenyl ring of an azobenzene arm and the carbon atoms of the ring from the neighboring star) beyond dispersion, which further links the stars into columns. These contacts have the largest fraction of 13.3% from the intermolecular interactions in the crystal. The separation of these contacts is 2.57 $\overset{˚}{A}$ (Figure 4c).

A very small fraction of close contacts making up only <1% of the surface occurs between two hydrogen atoms of the phenyl ring at the star periphery with distances of 2.04 or 2.30 $\overset{˚}{A}$. This type of close contact is on the spikes of the fingerprint plots as well (Figure 4d). The N⋯H, C⋯C, N⋯N contacts are marked as blue, meaning that these atoms are relatively far from each other in the crystals.

There are two important conclusions from the Hirshfeld surface analysis. First of all, the visual inspection of these charts shows the molecular asymmetry in terms of interactions with the surrounding molecules in the crystal. Secondly, the strongest interactions are realized along the stack due to the hydrogen bonds and C–H⋯π contacts. The last stabilizing interaction contributes to the lateral coupling between the columns.

The considered intermolecular interactions only qualitatively describe the forces holding together the ordering structures. Our next task is the quantification of the interactions. Partially, this has been solved recently by us [29] for the same object of the study. In that article, we have shown that the binding along the column is much stronger (up to a factor of six) than the intercolumnar coupling. The next section will be focused on the columnar stacks (Figure 1b,e) in order to establish which self-assembly mechanism the azo–BTA stars obey.

### 2.3. On the Cooperative Nature of Self-Assembly

The noncovalent association into one-dimensional columns is the first step in the appearance of complex supramolecular structures. Discovering the factors that affect the growth from a single azo–BTA star to the stacks and columns is a step forward to understanding the multi-structured morphologies found experimentally. One of these factors is the assembly mechanism, which is determined by the dependence of the association constant for monomer addition (in our case, azo–BTA star) on the assembly size *N* [56]. Here, the growth is isodesmic if the binding of each new monomer to the growing chain is independent of *N*, and cooperative if the binding shows enhanced propensity for monomer addition as *N* increases [44].

Our working hypothesis is that azo–BTA stars gather together cooperatively and that this growth in binding strength is due to electronic effects. According to literature data, these effects are manifested primarily based on pairwise electrostatic interactions, including long-range dipole-dipole interaction, non-pairwise short-range polarization, and resonance-assisted hydrogen bonding, and caused by the redistribution of the electron density along the column. As descriptors of cooperativity, the following features are analyzed as functions of *N* or depending on the position of the star in the stack: binding energies, the length of hydrogen bonds, and macrodipoles. These descriptors have been used by many researchers for the classification of the self-assembly mechanisms in BTA-containing systems [44,61,64,67]. The stacks of azo–BTA stars are analyzed using data sets from molecular dynamics (MD) simulations [29] and from experiments [17] applying density functional theory and classical modelling techniques.

**Figure 5:**
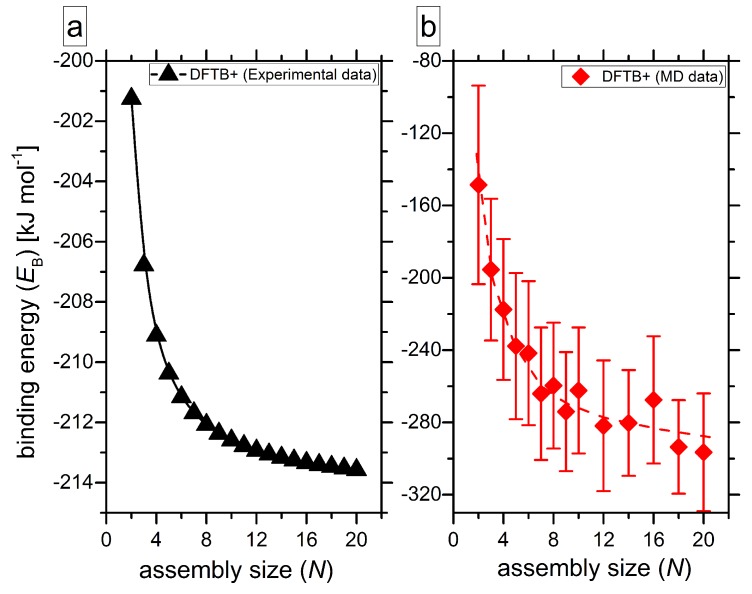
Binding energy *E*_B_ per pair as a function of assembly size *N* calculated for the star stack from the experimental crystal cell [17] (**a**) and for the columns modelled in MD simulations [29] (**b**). The dashed red line on panel (**b**) guides for the eye.

In Figure 5, we compare the binding energies per pair for azo–star columns of various length from experiments, i.e., for the stack shown in Figure 1e [17] (panel a), and modelled in MD simulations (panel b). Both data sets show an obvious *N*-dependence. This behaviour is analogous to results reported earlier [44,61,64,67]. The interaction strength increases with the number of molecules, proving that the formation of longer columns is energetically increasingly favorable, or in other words cooperative, as compared to shorter associates. On both panels, the binding energy decreases toward an asymptotic value for the infinitely long supramolecule. The values for the stacks with *N* = 20 are comparable to each other with −213 and −296 kJ mol^−1^ for Lee’s column (Figure 1e) and stacks from our recent publication [29], respectively. Here, it is important to underline that for such long columns of molecules with an all-atom representation of the star, the self-assembly rules are analyzed for the first time. In previous works, the objects of the simulations were either very short columns, or coarse-grainedd modelled stacks.

**Figure 6:**
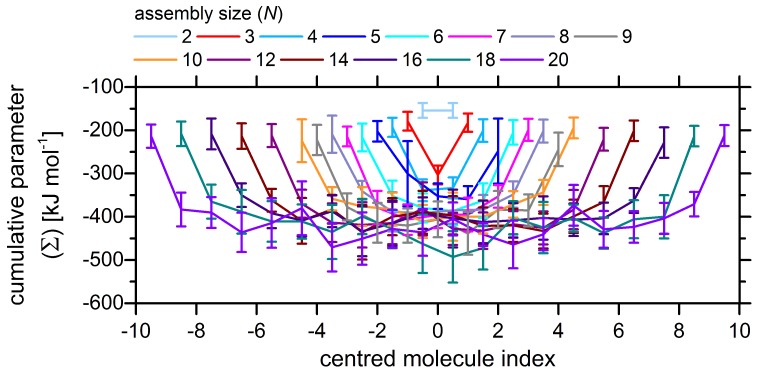
Cumulative parameter Σ calculated for each molecule in a stack depending on its position along the column of size *N*.

To rationalize the origin of this cooperative effect inherent to the longer azo–BTA stacks, the cumulative parameter Σ is calculated (Figure 6). First, the data from this plot indicate that the local interactions become stronger as the stack grows: The values for longer stacks are systematically shifted to more negative energies. This is a result of the binding to a larger number of molecules that are located beyond the nearest neighbors. Correspondingly, the circumstance that the outer stars only have binding partners to one side, leads to a weakening of their attachment. Hence, we observe approximately halved energy values when comparing the outer stars to centrally located molecules within the same cluster. Secondly, the coupling closer to the middle of the column is stronger than the interactions near the edges of the stack. Consider, however, that the irregular shape of curves in Figure 6 could hint at weak points and irregularities in the cluster themselves caused by effects coming from solvent molecules and thermal motion simulated explicitly in MD runs. The mentioned irregularities are locations where a cluster may be destabilized in ensuing photoisomerization events when the clusters are exposed to light.

**Figure 7:**
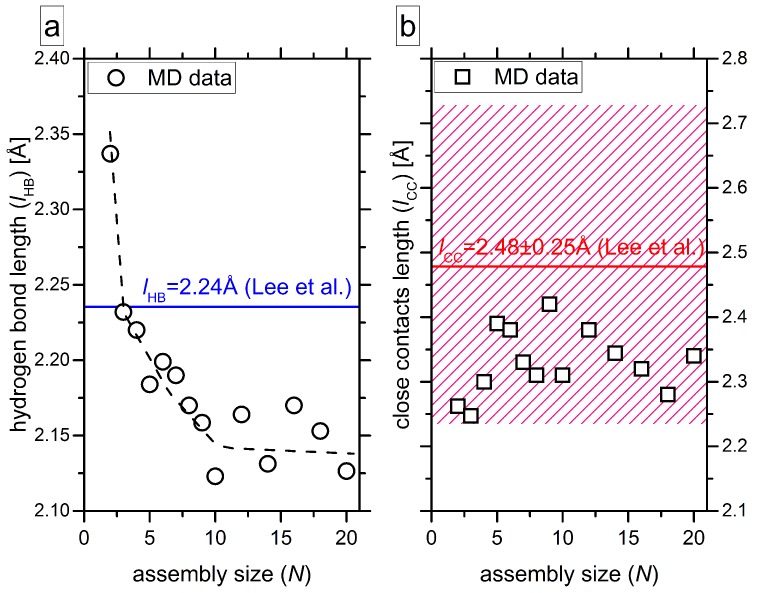
Average length of the intermolecular hydrogen bonds *l*_HB_ (**a**) and average length of the close contacts *l*_CC_ (**b**) as functions of assembly size *N*. The MD data from ref. [29] are taken for the analysis. The blue line in panel (**a**) shows the length of the hydrogen bonds as measured from the crystal cell studied by Lee et al. [17]. The dashed black line on panel (**a**) guides for the eye. The red line in panel (**b**) shows an average value for the close contacts as measured from the crystal cell studied by Lee et al. [17] and the red hatched field on the graph depicts the error bar above and below the average value.

The next feature pointing out the cooperativity of the self-assembly is the shortening of the hydrogen bonds upon stack growth (Figure 7a). Such a behavior has been observed in computational studies of smaller BTA-cored molecules [44,61]. In contrast to this, the close contacts do not show any clear dependence on the assembly size, and they cannot be considered as one of the descriptors of cooperativity (Figure 7b). As follows from Table 1, the strength of hydrogen bonding also changes depending on the position inside the stack. Although the values do not give a monotonic dependence, the trend is clearly seen: The shortest hydrogen bonds are found close to the center of the stack. Similar conclusions have been drawn in a computational study by Albuquerque et al. [61].

**Figure 8:**
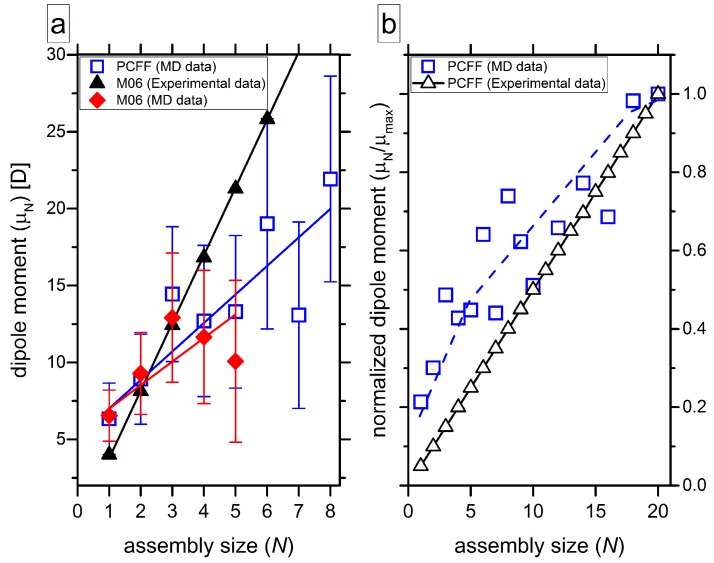
Average dipole moment $\mu_{N}$ (**a**) and normalized dipole moment $\mu_{N}$/$\mu_{\max}$ (**b**) as functions of assembly size *N*. The results shown on both panels as triangles refer to the structural data from ref. [17]. The other two data sets (red rhombi and open blue squares) are taken from snapshots of the MD trajectories [29]. The dashed blue line on panel (**b**) guides for the eye. In panel (**a**), error bars are depicted for the two MD data sets.

To examine the long-range dipole–dipole interactions, the average dipole moment $\mu_{N}$ and normalized macrodipole along the stacking direction $\mu_{N}$/$\mu_{\max}$ are calculated (Figure 8). The latter one is defined as the ratio of the electric dipole moment of an *N*-mer to the largest value of a dipole moment observed in the calculations, as described by Korlepara et al. [64]. In our calculations, $\mu_{\max}$ corresponds to *N* = 20. The dipole of the stack progressively grows (Figure 8a) due to the formation of hydrogen bonds, which requires the distortion of the planarity in the BTA-core: Weak intermolecular contacts force the rotation of the amide groups so that they do not remain in the plane of the benzene ring anymore. Most vividly it is seen for the stack from the crystalline structure [17]: The molecules have identical geometries, the dihedral angle Θ between the benzene core and amide group is around 30° for HB-bonded arms and ca. 8° otherwise, and the polar C = O groups point in the same direction increasing the long-axis component of the dipole.

A growing dipole of the stacks simulated in MD is predicted using different methods. However, the scattering of the data is substantial, which is related to a large number of molecular geometries that are realized at room temperature in computer simulations. For example, the mean value of the dihedral angle is Θ = 32.1 ± 17.8° for dimers. The carbonyl groups point in the same direction most of the time but not always, since thermal motion affects the molecular conformations. The values of the macrodipoles (Figure 8b) grow as well, which is in line with findings of other theoretical studies [61,67]. Thus, the amplification of electrostatic interactions along the stacks is the main factor leading to stronger hydrogen bonding between azo–BTA stars governing the cooperative growth of ordered columns.

## 3. Conclusions

Employing a number of computer simulation techniques, we have analyzed the intermolecular interactions between the azo–BTA stars in columns in experimentally resolved crystals [17] and as obtained in our recent molecular dynamics study [29]. Our results suggest that during the formation of ordered phases, these molecules prefer to build 1D columns with the equilibrium distance between the star cores of 3.6 $\overset{˚}{A}$, in which stars are held together via weak interactions. These noncovalent interactions include among others the hydrogen bonding, whose length shortens with increasing the column length.

The binding energy, which accounts for all the noncovalent through-space interactions, grows with increasing size of the aggregate, as our calculations demonstrate. This phenomenon called cooperative self-assembly is proven for azo–BTA stars in aqueous phases. The simulations clearly attribute the cooperativity of the self-assembly to the development of a macrodipole, in other words, to the redistribution of the electronic density. This, in turn, enhances the strength of the hydrogen bonds, and subsequently the intermolecular coupling. We find that the predictions of binding energies and the molecular coordinates in the stacks coincide with data for the photosensitive azobenzene stars investigated in seminal works by Lee et al. [17,18,47].

The next steps of this work could include, for instance, the calculation of the cooperativity factor σ [45] in order to place the modelled azobenzene star on the cooperativity scale for BTA-cored derivatives. Another interesting topic is related to the recently published paper by Bochicchio et al. [68] on the light-induced evolution of an azobenzene-containing supramolecular tubule in view of our data showed in Figure 6, where the defects are characterized quantitatively for the azo–BTA column. Finally, here we considered the stacks of azobenzenes in the dark, i.e., the stars in all-trans state. It would be interesting to elucidate the behavior of all-cis azo–BTA stars and the energetics of their self-assembly, since, as we showed recently, the stacks made by all-cis azobenzene stars maintain their clusters upon light stimulus, even though their shape is more disordered, as compared to the stacks in the dark.

## 4. Materials and Methods

### 4.1. Objects of the Study and Their Models

In this paper, we consider three objects. The first one is a dimer constructed by doubling the all-trans azo–BTA star shown in Figure 1a in its optimized geometry, as obtained in our previous study [14]. The second one is the column of *N* = 2–20 stars formed in MD simulations in a recent paper [29], where *N* is the assembly size. One example with *N* = 20 is depicted in Figure 1b. Finally, the third object is the crystal of azo–BTA star experimentally characterized by Lee et al. [17] (Figure 1c–e). Here, we study the crystal itself, as well as the molecular stacks obtained by multiplying the original unit cell in *b*-direction 2–20 times. In this case, we get the columnar assemblies of the same size *N* as in MD simulations, but having identical molecular geometries, as revealed in experiments in ref. [17]. In all the cases, we use a full-atomistic representation of the stars, whereas the aqueous environment is modelled applying two schemes: In density functional theory (DFT) and density functional based tight-binding (DFTB+) calculations, the effects coming from the solvent are taken into account via the polarizable continuum model [69], whereas for the simulation of assemblies in molecular dynamics, water is simulated explicitly.

The columns, which vary in their size between two and 20 molecules, are manually assembled for the subsequent MD simulations. The columns are built by repeatedly placing centrally aligned stars at a distance of 5.0 $\overset{˚}{A}$ from each other. Later, during the minimization process and MD simulations, the molecules find their preferred distances. Using the Forcite module of Materials Studio [70], the preformed clusters are placed into a rectangular box with side lengths of *L*_x_ = *L*_B_ = 81 $\overset{˚}{A}$ and *L*_z_ = 140 $\overset{˚}{A}$ for the initial setup. Subsequently, to achieve an overall density of 1.0 g cm^−3^, a total of 28978 water molecules are randomly and homogeneously distributed to solvate the columnar cluster. Finally, the configurations are transferred to LAMMPS to simulate each system for 10 ns. All simulations are performed in the NPT ensemble (Nos*é*–Hoover barostat and thermostat) at the temperature *T* = 298 K and pressure *P* = 1 atm while using a time step of 1 fs. More details on the MD protocols, polymer consistent force field (PCFF) parameters, etc. can be found in ref. [29].

### 4.2. Computation of the Binding Energy Diagrams and the Binding Energy as a Function of Assembly Size N

The definition of the binding energy is given by Equation (1). The diagrams for the dimers (Figure 2 and Figure 3) are obtained using the M06-L/aug-cc-pVTZ functional [71] and basis set with a semi-empirical counterpoise-type correction for basis set superposition error, as implemented in Gaussian 09 [72]. The binding of larger stacks from either MD simulations or experimental data (Figure 5) is quantified applying density functional based tight-binding with parameter set mio-1-1 [73].

The cumulative parameter Σ (Figure 6) is calculated as a function of the molecule index *i* within the stack by using the group/group command of LAMMPS [74]. For each molecule in the column-shaped cluster the pairwise interactions with all the other azobenzene stars are calculated, excluding any self-interactions within molecule *i*. More precisely, considering a molecule with index *i*, all pairwise Coulombic and Lennard–Jones energy contributions coming from the *N*-1 surrounding molecules (with indices ≠ *i*) are summed up to Σ(*i*). The parameter Σ(*i*) therefore describes the strength of binding of the *i*-th azo star with the entire rest of the cluster. Please note, that the sum of Σ(*i*) over all molecules *i* in the stack is clearly not equal to the binding energy $E_{B}$ of a cluster as defined above. This occurs, since for any molecules *i* and *j* in the stack there are reappearing energy contributions to Σ(*i*) or Σ(*j*), respectively, for example from another nearby molecule *k*. The results for the cumulative parameter are obtained by averaging over an ensemble of three independent simulations of 10 ns for each cluster size.

### 4.3. Hirshfeld Surface Analysis of the azo–BTA Star Crystal

To more closely examine the intermolecular interactions in the crystals of azo-stars experimentally characterized by Lee et al. [17], the Hirshfeld surfaces [65,66] are generated using CrystalExplorer (Version 3.1) [75]. This method has been successfully utilized to characterize aminoazobenzene polymorphs [76], to investigate the intermolecular interactions in azobenzenes revealing photoinduced crystal–melt transition [77], and to explain the molecular origin of the color changes of guest signaling azobenzene [78,79], to name a few.

The Hirshfeld surface is a three-dimensional isosurface effectively determining the volume of the molecule in the crystal. The surface itself is defined so that for each point on this surface, one-half of the electron density is due to the spherically averaged noninteracting atoms comprising the molecule, and the other half is due to those comprising the rest of the crystal [66]. The quantity which is mapped onto the surface is $d_{\mathrm{norm}}$ (normalized contact distance), which is the negative or positive sum of $d_{i}$ and $d_{e}$ when the contacts are shorter or longer than the sum of the van der Waals radii of the interacting atoms, respectively:

(2) $$d_{\mathrm{norm}}=\frac{\left(d_{i}-r_{i}^{\mathrm{vdW}}\right)}{r_{i}^{\mathrm{vdW}}}+\frac{\left(d_{e}-r_{e}^{\mathrm{vdW}}\right)}{r_{e}^{\mathrm{vdW}}}.$$

The fingerprint plots (*d*_e_-*d*_i_ diagrams) are constructed using $d_{e}$ and $d_{i}$ distances from the Hirshfeld surface to the nearest atom outside (external) and inside (internal) the surface, correspondingly. These plots provide a summary of the intermolecular contacts in the crystal.

### 4.4. Quantification of Hydrogen Bonds, Close Contacts and Macrodipoles

The length of the hydrogen bond *l*_HB_ is considered as a separation between the hydrogen and oxygen atoms of the amide groups from the neighboring stars in the stack applying the structural definitions of the bond suggested by Luzar and Chandler [80]. The close contacts *l*_CC_ are defined in terms of the sum of the specified atomic radii. Finally, the dipole moments $\mu_{N}$ of the stars and stacks with *N*≤ 8 are calculated using density functional theory with M06-L/aug-cc-pVTZ hybrid functional, as justified by Zawada et al. for hydrogen-bonded molecular chains [81]. For comparison and for larger aggregates, the point-like charges on atoms from PCFF parametrization are used to calculate the normalized dipole moments as $\mu_{N}$/$\mu_{\max}$, where $\mu_{\max}$ is the maximal value of the dipole molecule of the stack with *N* = 20. All the aforementioned properties are analyzed using the Forcite module of BIOVIA Materials Studio [70].

## Figures and Tables

**Table 1:** Mean intermolecular hydrogen bond length *l*_HB_ in $\overset{˚}{A}$ as a function of the position inside the stack. The indices *i* and *j* of *N*_*i*–*j*_ (1–2, 2–3, etc.) denote the location of the hydrogen bonds, i.e., the index numbers of hydrogen-bonded molecules in the stack of size *N* = 20.

*N*_*i*–*j*_	*N*_1–2_	*N*_2–3_	*N*_3–4_	*N*_4–5_	*N*_5–6_	*N*_6–7_	*N*_7–8_	*N*_8–9_	*N*_9–10_	
*l*_HB_	2.34	2.14	2.16	2.22	2.15	1.93	2.15	2.00	2.02	
*N*_*i*–*j*_	*N*_10–11_	*N*_11–12_	*N*_12–13_	*N*_13–14_	*N*_14–15_	*N*_15–16_	*N*_16–17_	*N*_17–18_	*N*_18–19_	*N*_19–20_
*l*_HB_	2.18	2.08	2.17	2.05	1.99	2.04	2.11	2.08	2.21	2.25

## Abbreviations

The following abbreviations are used in this manuscript:

BTA	Benzene-1,3,5-tricarboxamide
azo–BTA star	Three-arm star-shaped molecule with BTA core and azobenzene arms
DFT	Density functional theory
DFTB+	Density functional based tight-binding
MD	Molecular dynamics
PCFF	Polymer consistent force field
LAMMPS	Large-scale atomic/molecular massively parallel simulator
